# A Facile Approach to Tune the Electrical and Thermal Properties of Graphene Aerogels by Including Bulk MoS_2_

**DOI:** 10.3390/nano7120420

**Published:** 2017-12-01

**Authors:** Feng Gong, Xiongxiong Liu, Yunlong Yang, Dawei Xia, Wenbin Wang, Hai M. Duong, Dimitrios V. Papavassiliou, Ziqiang Xu, Jiaxuan Liao, Mengqiang Wu

**Affiliations:** 1 School of Energy Science and Engineering, University of Electronic Science and Technology of China, Chengdu 611731, China; 18215565716@163.con (X.L.); m15680731552@163.com (Y.Y.); davidxia97@163.com (D.X.); bingo_uestc@163.com (W.W.); nanterxu@uestc.edu.cn (Z.X.); jxliao@uestc.edu.cn (J.L.); 2 Department of Mechanical Engineering, National University of Singapore, Singapore 117576, Singapore; mpedhm@nus.edu.sg; 3 School of Chemical, Biological and Materials Engineering, University of Oklahoma, Norman, OK 73019, USA

**Keywords:** graphene aerogel, molybdenum disulfide, tunable thermal conductivity, electrical conductivity, energy storage

## Abstract

Graphene aerogels (GAs) have attracted extensive interest in diverse fields, owing to their ultrahigh surface area, low density and decent electrical conductivity. However, the undesirable thermal conductivity of GAs may limit their applications in energy storage devices. Here, we report a facile hydrothermal method to modulate both the electrical and thermal properties of GAs by including bulk molybdenum disulfide (MoS_2_). It was found that MoS_2_ can help to reduce the size of graphene sheets and improve their dispersion, leading to the uniform porous micro-structure of GAs. The electrical measurement showed that the electrical conductivity of GAs could be decreased by 87% by adding 0.132 Vol % of MoS_2_. On the contrary, the thermal conductivity of GAs could be increased by ~51% by including 0.2 vol % of MoS_2_. The quantitative investigation demonstrated that the effective medium theories (EMTs) could be applied to predict the thermal conductivity of composite GAs. Our findings indicated that the electrical and thermal properties of GAs can be tuned for the applications in various fields.

## 1. Introduction

Over the past few years, graphene has exerted tremendous impacts in diverse fields [1,2]. It has considerable potential for applications in microelectronic devices and energy storage devices due to its preeminent electrical, thermal, mechanical and optical properties [3,4,5,6,7]. These unique properties of graphene result from its two-dimensional features. Nevertheless, there are few reliable approaches to handling such an atomically-thin material, which hampers its device-grade properties and applications. Recently, graphene aerogels (GAs), a type of self-assembly of the bulk materials of graphene, have attracted much attention because of their outstanding nature, such as ultrahigh surface area and ultralow bulk density [8,9]. 

GA represents a new form of monolithic structure with high porosity, low density and superb conductivity, which can serve as a promising candidate in various areas [10,11,12]. Moreover, GAs can be readily prepared via a facile hydrothermal method [13]. Wu et al. [14] prepared three-dimensional (3D) nitrogen-doped graphene aerogel (N-GA) to support Fe_3_O_4_ nanoparticles (Fe_3_O_4_/N-GAs) for the oxygen reduction reaction (ORR). Due to their porous structure and high surface area, Fe_3_O_4_/N-GAs exhibited better ORR performances and durability than the commercial Pt/C catalyst. Kim et al. [15] obtained GAs with an ultrahigh surface area (up to 3290 m^2^/g) for supercapacitors. The GAs showed both high volumetric (100 F/cm^3^) and gravimetric (174 F/g) specific capacitance. Besides, Jiang et al. [16] reported MoS_2_-graphene hybrid aerogels with controllable porosity for lithium-ion batteries. The hybrid aerogels displayed a high reversible capacity up to 1200 mAh/g, as well as sterling rate capability. 

Besides hydrothermal methods, several other techniques were also been utilized to fabricate GAs and related materials. For instance, Chen et al. [17] synthesized 3D graphene foams (GFs) through a template-directed chemical vapor strategy, where nickel foams were employed as templates. The GFs exhibited considerably high electrical conductivity and demonstrated a great potential to obtain GF/polymer composites for flexible and stretchable conductors. More recently, Samad and coworkers developed a novel two-step technique to create GFs with tunable density, superior electrical conductivity and moldable structure [18]. When combining GFs with polymer, the as-prepared composites displayed ameliorated mechanical properties, suitable for high and low pressure applications, as well as strain sensing. 

The outstanding performance of GAs in the aforementioned applications may be partially ascribed to their high electrical conductivity [11]. Unfortunately, most GAs possess very low thermal conductivity (~0.01 W/mK), five orders of magnitude lower than that of graphene nanosheets (~5000 W/mK) [19]. Guo et al. [9] prepared graphene/carbon aerogels by sol-gel polymerization and ambient pressure drying. The obtained aerogels attained a thermal conductivity of 0.028–0.08 W/mK. Wicklein et al. [20] used a free-casting method to prepare cellulose nanofibers (CNF)/graphene oxide (GO)/sepiolite nanorods (SEP) hybrid aerogels. The hybrid aerogels displayed a thermal conductivity of 0.015 W/mK. Xie et al. [21] synthesized GAs through a modified Hummer’s method and freeze drying. Under vacuum and low temperature, the GAs demonstrated ultralow thermal conductivity (~2.0 × 10^−4^ W/mK). The poor thermal conductivity of GAs may contribute to possible applications in thermal insulation and heat protection fields. However, in energy storage, like lithium ion batteries, the low thermal conductivity of GAs may deteriorate the heat dissipation from the devices, causing serious safety problems. Thus, refining the thermal transport properties of GAs may broaden their applications in energy storage fields. 

In recent years, 2D transition metal dichalcogenides (MoS_2_, WS_2_) have been combined with graphene for advanced functional materials. Worley et al. [22] prepared monolithic WS_2_, MoS_2_ and MoS_2_/graphene aerogels via thermal decomposition of freeze-dried precursors. The WS_2_ and MoS_2_ aerogels showed an ultralow density, and the MoS_2_/graphene aerogels manifested large surface areas, desirable electrical conductivity and excellent activity in the hydrogen evolution reaction. Fang et al. [23] synthesized MoS_2_/graphene heterostructures for lithium ion battery, where an initial specific capacity of ~1800 mAh/g was achieved, over two-times higher than that of pure MoS_2_. The MoS_2_/graphene hybrid also displayed excellent cycling and rate properties. Li and coworkers also prepared WS_2_/reduced graphene oxide (rGO) composites for lithium ion battery [24]. The rGO could enhance both the electrochemical properties and chemical stability of WS_2_, inducing a better performance of the composite as anode materials [24]. 

Nonetheless, there are few studies that systematically and quantitatively investigate the electrical and thermal properties of GAs: the existing research works solely focused on either electrochemical or thermal properties. In this work, based on the above studies, we readily used MoS_2_ to adjust the electrical and thermal transport properties of GAs. GAs were prepared from the graphene oxide (GO) solution via a facile hydrothermal method and freeze drying, while GO was obtained from the raw graphite powers through the modified Hummers’ method. The obtained GAs showed desirable mechanical and electrical properties, but poor thermal transport property. Via the addition of MoS_2_ into GAs, the thermal conductivity of the hybrid aerogels could be elevated. The effective medium theory (EMT) was applied to investigate the enhancement in thermal conductivity of GAs. The predicted thermal conductivity values from the EMT were in excellent agreement with the experimental data. The mechanisms by which MoS_2_ can affect the electrical and thermal conductivity of GAs were discussed in detail by taking into account the microstructures of GAs. These quantitative findings may shed light on the strategy to adjust electrical and thermal properties of GAs for diverse applications. 

## 2. Experiment

### 2.1. Materials

The chemicals used in the experiments were all purchased from Sinopharm Chemical Reagent Corp. (Shanghai, China), and they are listed as below:

Graphite powder, sodium nitrate (NaNO_3_), potassium permanganate (KMnO_4_), concentrated sulfuric acid (H_2_SO_4_, 98%), hydrogen peroxide (H_2_O_2_, 30%), hydrochloric acid (HCl), polyvinyl chloride (PVC), ethylenediamine (EDA), ammonium hydroxide (NH_3_·H_2_O), molybdenum disulfide (MoS_2_), iso-propyl alcohol (IPA) and ethanol. 

### 2.2. Synthesis of Graphene Aerogels 

Graphene oxide (GO) aqueous solution was obtained from the raw graphite powders by a modified Hummers’ method [25]. With the chemical reduction of the GO solution with ethylenediamine (EDA) at 120 °C for 12 h, graphene hydrogels were synthesized. Then, graphene aerogels (GAs) were prepared by freeze-drying the graphene hydrogels for another 12 h. GAs from different GO concentrations (2, 3, 4 and 6 mg/mL) were fabricated for comparison. To synthesize MoS_2_/graphene hybrid aerogels, 20 mg of MoS_2_ powder were dispersed in 10 mL of IPA/water (45 vol %) mixed solvent in a 50-mL flask, and the solution was sonicated for 30 min. Subsequently, a certain amount of GO suspension (4 mg/mL) was added into the above solution and continually sonicated for another 30 min, followed by hydrothermal reduction at 120 °C for 12 h. The above hydrogels became aerogels through the aforementioned freeze-drying.

### 2.3. Characterization 

X-ray diffraction (XRD) analysis was conducted on a Bruker-D8 Advanced X-ray Diffractometer (Karlsruhe, Germany) (Cu Kα radiation: λ = 1.5406 Å) with a scanning speed of 5 deg/min. Scanning electron microscope with energy dispersive spectroscopy (SEM-EDS) was measured using JSM-65900LV, JSM-7500F, JEOL, (Tokyo, Japan). Brunauer–Emmett–Teller (BET) surface area was tested on Micrometrics Instrument Corp. ASAP-2020M (Norcross, GA, USA). The electrical conductivity was measured by a home-made device with a programmable SC power supply (INTERLOCK IPD-6006SLU, Chengdu, China). Thermal conductivity of aerogels was measured according to the transient hot-wire method (Hot Disk TPS 2500S, Uppsala, Sweden) [26]. 

## 3. Results and Discussion

### 3.1. Macro-Scale Morphology and Mechanical Property of Graphene Aerogels 

**Figure 1:**
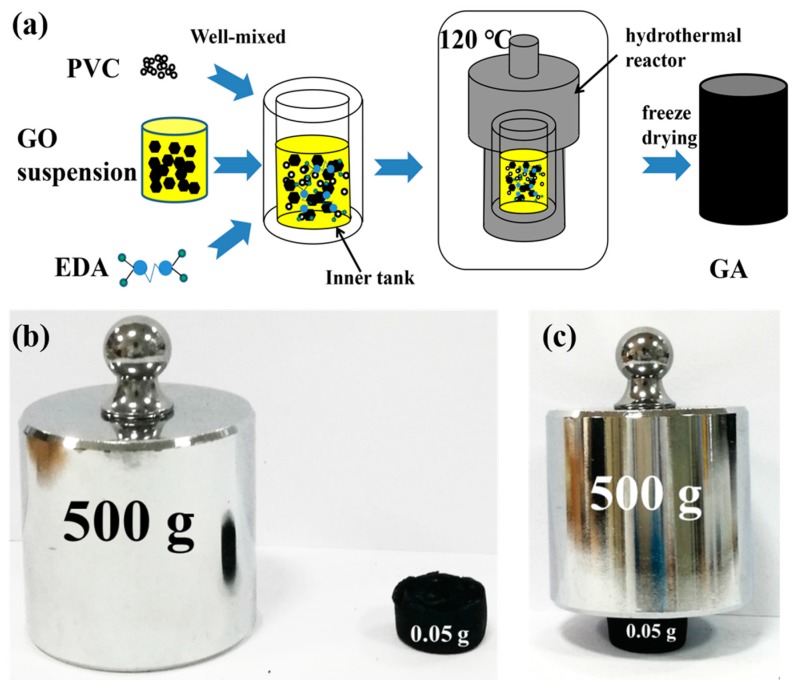
Macro-scale morphology and demonstration of the mechanical strength of graphene aerogels (GAs). (**a**) The schematic plot of the self-assembly process of GAs from GO solution; (**b**) the size and weight contrasts of a 500-g weight and the GA obtained from the 2-mg/mL GO solution; (**c**) the GA can easily support the 500-g weight without destroying its structure, showing excellent mechanical strength. Composite GA with MoS_2_ has a similar cylindrical shape as pure GA. However, composite GA showed volume shrinkage compared with pure GA, which is discussed in the following sections.

Figure 1a shows the schematic plot of the synthesis process of graphene aerogels (GAs). One can easily follow the facile method to acquire the 3D bulk materials of graphene. During our study, we found that the minimum concentration of graphene oxide (GO) for the self-assembly of GAs was 1.5 mg/mL. Therefore, the solutions with GO concentrations of 2, 3, 4, and 6 mg/mL were used to synthesize GAs in our study. The volume of the final GAs increased with heightened GO concentration. The final product of GAs was a black cylinder, as shown in Figure 1b,c. The cylindrical shape of GAs was due to the cylindrical hydrothermal reactor. One can also obtain other shapes of GAs by using different reactors [25,27,28]. The acquired GAs showed a very low density (~0.015 g/cm^3^) and excellent mechanical strength, as exhibited in Figure 1c. The GA with a weight of 0.05 g can easily support a 500-g weight, revealing a strong mechanical strength. It should be pointed out that the maximum standardized weight in our lab is only 500 g, and the mechanical properties of GAs will be characterized in detail in our further study. 

### 3.2. Micro-Scale Morphology of Pure GA and Composite GA (with MoS_2_)

**Figure 2:**
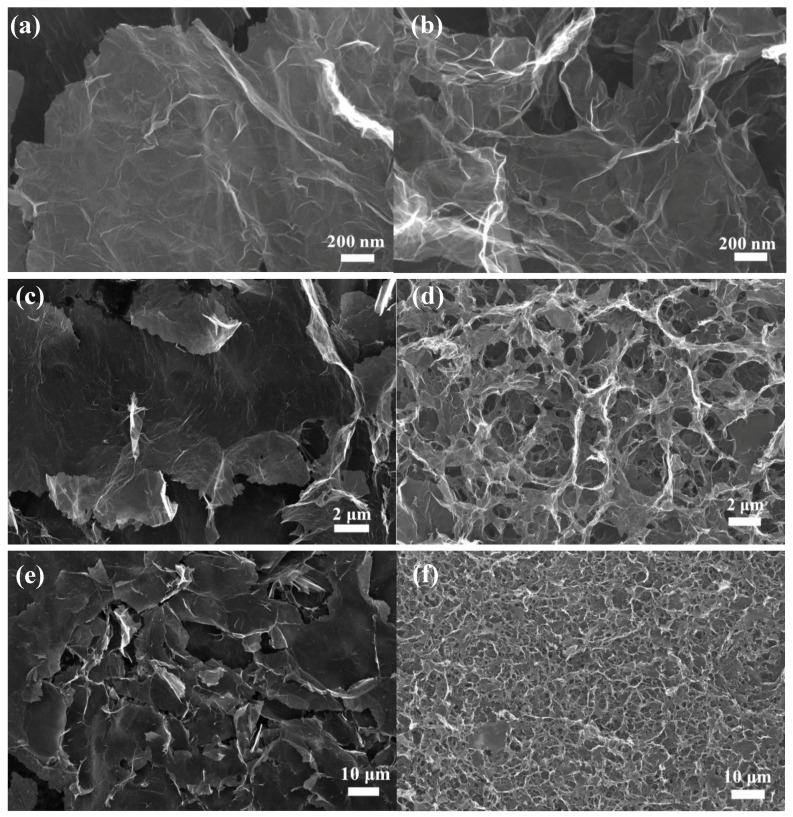
Scanning electron microscope (SEM) images of pure GA (**a**,**c**,**e**) and composite GA with MoS_2_ (**b**,**d**,**f**). The GO concentration for both GAs was 4 mg/mL, and the concentration of MoS_2_ was 5 wt %. The BET (Brunauer–Emmett–Teller) surface area test showed that the specific surface areas for the above pure and composite GAs were 182.8 and 91.8 m^2^/g, respectively.

Figure 2a–f presents the micro-scale morphologies of pure GA and the GA with MoS_2_. The concentrations of GO for pure GA and composite GA were both 4 mg/mL, and the addition of MoS_2_ was 5 wt %. As shown in Figure 2, pure GA and composite GA exhibited rather different micro-scale morphologies. Graphene sheets in pure GA are much larger than those in composite GA, as illustrated in Figure 2a,b. This may be assigned to the extra sonication after adding MoS_2_ for composite GAs, which greatly reduced the size of graphene sheets. 

Moreover, the pore size in composite GA was also notably decreased by including MoS_2_, as displayed in Figure 2c,d. The reduced pore size induced not only the volume shrinkage of composite GA, but also a lower specific surface area of composite GA (~91.8 m^2^/g) than that of pure GA (~182.8 m^2^/g). However, comparing with pure GA, composite GA shows a more uniform porous structure with smaller pores, observed from Figure 2e,f. This unique micro-scale morphology may benefit the thermal transport properties of composite GA. The different brightness of SEM images roughly show the lower electrical conductivity of composite GA compared to pure GA, which will be further demonstrated in the following section.

**Figure 3:**
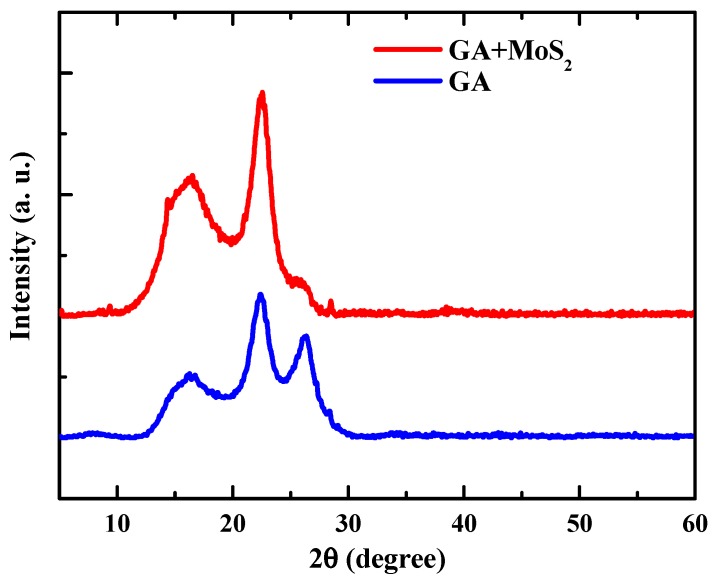
XRD patterns of composite graphene aerogel and pure graphene aerogel.

Figure 3 presents the XRD patterns of pure GA and composite GA. The three main peaks in the XRD patterns of pure GA are located at 2θ = 16.3°, 22.3° and 26.4°, which correspond to the peaks of expanded graphite [29], reduced graphene oxide [30] and graphite [25], respectively. The existence of expanded graphite flakes and graphite flakes was also corroborated by the SEM images, as shown in Figure 2a,c,e. In the XRD patterns of composite GA, the peaks of expanded graphite (16.3°) and reduced graphene oxide (22.3°) still remain, but the graphite peak (26.4°) is significantly reduced. This may be due to the reduced size of graphite flakes by adding MoS_2_. The peak at 14.4° indicates the existence of bulk MoS_2_ [31], which means that the MoS_2_ was not dispersed as monolayers, but as multilayers. 

### 3.3. Electrical Property of Pure GA and Composite GA 

**Figure 4:**
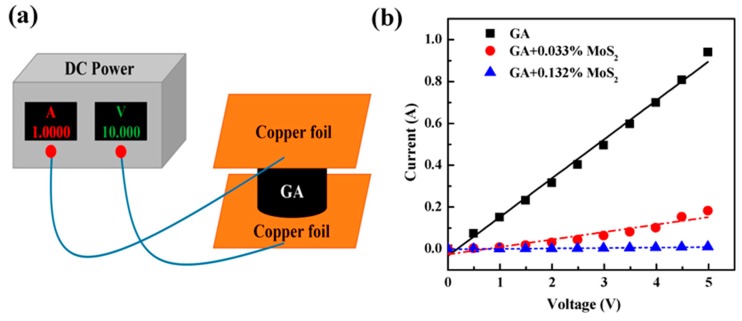
Electrical property of composite GAs: (**a**) The illustration of the homemade device to measure the electrical conductivity of GAs. The DC power can supply DC voltages and measure the current through the circuit. Silver paste was used between the copper electrode and GA sample to reduce the contact resistance. The current through GA samples was recorded under different voltages to obtain the electrical conductivity of the GA sample, as shown in (**b**).

To investigate the electrical properties of pure GA and composite GA, their electrical conductivity was measured and calculated by a homemade device (Figure 4a). A direct current (DC) power supply was used to supply DC voltages (*V*). Two copper foils were used as electrodes to connect the DC power supply and GA samples. To reduce the contact resistance between GA and the copper electrodes, silver paste was applied between them. The current (*I*) was recorded under different voltages, as shown in Figure 4b. Then, the electrical conductivity of the GA sample can be obtained as below:

(1) $$\sigma =\frac{IL}{VA}$$

where *L* is the length of GA samples and *A* is the cross-section area of GA samples. 

As shown in Figure 4b, at the same voltage, the current through GA samples dramatically decreased with the increase of MoS_2_ addition. This indicated that MoS_2_ lowered the electrical conductivity of GAs. According to Equation (1), the electrical conductivity of pure GA, the GA with 0.033 vol % MoS_2_ and the GA with 0.132 vol % MoS_2_ was calculated to be 13.6, 4.1 and 1.8 S/m, respectively (Table 1). The significant decrease in electrical conductivity of GA by adding MoS_2_ may be attributable to the following reasons:

(1) MoS_2_ has rather poorer electrical conductivity (~3 S/m [32]) compared to that of graphene (~100 S/m [33]). When added into GAs, MoS_2_ acted as an insulator to block electron transfer in GAs, thus inducing a lower electrical conductivity. 

(2) MoS_2_ could reduce the size of graphene sheets. The smaller graphene sheets will have an increased scattering probability of electrons, which also results in a lower electrical conductivity of composite GAs. 

Here, we suggested an effective approach to reduce the electrical conductivity of GAs. This approach can be applied in electronic packaging materials, where higher thermal conductivity, but lower electrical conductivity of the packaging material is preferred. Specifically, it should be noted that the electrical conductivity of GAs is one of the key factors for their applications in energy storage devices. Fortunately, the reduced electrical conductivity of GAs in the present study is still comparable to those of GAs used for energy storage in other studies [13,27,39]. We believe that with the proper amount of MoS_2_, the energy storage and thermal transport properties of GAs can be enhanced without remarkably deteriorating their electrical conductivity. 

### 3.4. Thermal Transport Property of Pure GAs and Composite GAs 

**Figure 5:**
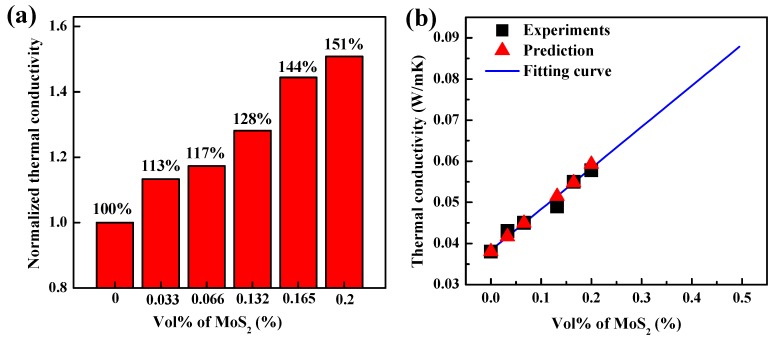
Thermal transport properties of composite GAs. (**a**) Normalized thermal conductivity of composite GAs with different volume factions of MoS_2_; (**b**) the calculated thermal conductivity of composite GAs from the effective medium theories (EMTs) showed an excellent agreement with the measured values. The fitting curve was plotted according to the obtained values from the EMTs.

The concentration range of GO was set as 2–6 mg/mL to study its effect on the thermal conductivity of the final GAs. It was found that the thermal conductivity of all GAs was around 0.04 W/mK, indicating that the GO concentration does not have an apparent impact on the thermal conductivity of GAs. With the inclusion of MoS_2_, the thermal conductivity of composite GAs was substantially increased, as shown in Figure 5a. After adding 0.033, 0.066, 0.132, 0.165 and 0.2 vol % of MoS_2_, the thermal conductivity of composite GAs was elevated by 13%, 17%, 28%, 44% and 51%, respectively. The enhancement in the thermal conductivity by MoS_2_ addition may be owed to the following mechanisms:

(1) MoS_2_ reduced the pore size and lowered the surface area in composite GAs, thus decreasing the probability of phonon scattering at air-graphene interfaces.
(2) The MoS_2_ may uniformly disperse at graphene-graphene interfaces to lower the interfacial thermal resistances, thus accelerating the transfer of heat among graphene.

To quantitatively investigate the thermal conductivity of composite GAs, the effective medium theories (EMTs) were applied to predict the thermal conductivity of composite GAs (*K*_eff_), which was expressed as [40]:

(2) $$K_{\mathrm{eff}}=\frac{K_{{\mathrm{MoS}}_{2}}[3K_{\mathrm{GA}}+2f(K_{{\mathrm{MoS}}_{2}}-K_{\mathrm{GA}})]}{(3-f)K_{{\mathrm{MoS}}_{2}}+fK_{\mathrm{GA}}+\frac{fR_{B}K_{\mathrm{GA}}K_{{\mathrm{MoS}}_{2}}}{H}}$$

where $K_{{\mathrm{MoS}}_{2}}$ and $K_{\mathrm{GA}}$ are the thermal conductivity of MoS_2_ and pure GA. f, $R_{B}$ and H are the volume fraction of MoS_2_, the interfacial thermal resistance and the thickness of MoS_2_, respectively. All the parameters used in the calculation are listed in Table 1. 

Here, pure GA was considered as the matrix, and the multilayered MoS_2_ was treated as the fillers. The prediction of the effective thermal conductivity (*K*_eff_) was conducted as follows: the interfacial thermal resistance, $R_{B}$, was varied to match the calculated *K*_eff_ with the measured *K*_eff_ of one composite GA (e.g., 0.066 vol % of MoS_2_). Then, the obtained $R_{B}$ was used to calculate the *K*_eff_ of other composite GAs. The estimated $R_{B}$ was 0.9 × 10^−8^ m^2^ K/W, which was consistent with the reported values [16,41,42,43,44,45]. As shown in Figure 5b, the calculated *K*_eff_ of composite GAs square well with the experimental values, indicating that the EMTs can be applied to successfully predict the *K*_eff_ of composite GAs. This may be because the EMTs can take into account the complex geometry of MoS_2_, as well as the interfacial thermal resistances between MoS_2_ and pure GAs. Using the obtained values, we can have the equation to predict the general *K*_eff_ of composite GAs, as plotted in Figure 5b as the fitting curve. 

## 4. Conclusions

In conclusion, we successfully modulated the electrical and thermal conductivity of GAs by utilizing MoS_2_. By adding 0.33~0.2 vol % of MoS_2_, the electrical conductivity of GAs can be modulated between 1.8 S/m and 13.6 S/m, and meanwhile, the thermal conductivity of GAs can be increased from 0.038 to ~0.058 W/mK (~51% enhancement). The effect of MoS_2_ addition on the electrical and thermal conductivity of GAs may be attributed to the reduced size and improved dispersion of graphene sheets, which decelerate the electron conduction, but accelerate the phonon conduction at the graphene/MoS_2_/graphene interfaces. The EMTs can be employed to predict the effective thermal conductivity of GAs by taking into account the complex morphology of MoS_2_ and the thermal resistance at MoS_2_-graphene interfaces. These findings proposed a facile approach to procure GAs with tuned electrical and thermal properties for the large-scale applications in electronic packing and energy storage fields. 

## Figures and Tables

**Table 1:** Parameters used to predict the effective thermal conductivity of composite GA by using the EMT and the measured electrical conductivity of composite GAs.

Parameter	Value
Thermal conductivity of pure GA $K_{\mathrm{GA}}$ (W/mK)	0.038
Thermal conductivity of MoS_2_ $K_{{\mathrm{MoS}}_{2}}$ (W/mK) [34,35,36,37]	15
Volume fraction of MoS_2_ *f* (%)	0–0.5
Thickness of the multilayered MoS_2_ (nm)	10
Interfacial thermal resistance $R_{B}$ (10^−8^ m^2^K/W)	0.9
Density of graphene (g/cm^3^) [38]	1.06
Density of MoS_2_ (g/cm^3^)	4.8
Density of air (g/cm^3^)	0.0012
Volume fraction of MoS_2_ (%)	Electrical conductivity (S/m)
0	13.6
0.033	4.1
0.132	1.8

## References

[1] Xia F., Farmer D.B., Lin Y.-M., Avouris P. (2010). Graphene Field-Effect Transistors with High On/Off Current Ratio and Large Transport Band Gap at Room Temperature. Nano Lett..

[2] Huang X., Qi X., Boey F., Zhang H. (2012). Graphene-based composites. Chem. Soc. Rev..

[3] Novoselov K.S., Geim A.K., Morozov S.V., Jiang D., Katsnelson M.I., Grigorieva I.V., Dubonos S.V., Firsov A.A. (2005). Two-dimensional gas of massless Dirac fermions in graphene. Nature.

[4] Balandin A.A., Ghosh S., Bao W., Calizo I., Teweldebrhan D., Miao F., Lau C.N. (2008). Superior Thermal Conductivity of Single-Layer Graphene. Nano Lett..

[5] Lee C., Wei X., Kysar J.W., Hone J. (2008). Measurement of the Elastic Properties and Intrinsic Strength of Monolayer Graphene. Science.

[6] Mak K.F., Sfeir M.Y., Wu Y., Lui C.H., Misewich J.A., Heinz T.F. (2008). Measurement of the Optical Conductivity of Graphene. Phys. Rev. Lett..

[7] Zhu Y., Murali S., Cai W., Li X., Suk J.W., Potts J.R., Ruoff R.S. (2010). Graphene and Graphene Oxide: Synthesis, Properties, and Applications. Adv. Mater..

[8] Qian Y., Ismail I.M., Stein A. (2014). Ultralight, high-surface-area, multifunctional graphene-based aerogels from self-assembly of graphene oxide and resol. Carbon.

[9] Guo K., Hu Z., Song H., Du X., Zhong L., Chen X. (2015). Low-density graphene/carbon composite aerogels prepared at ambient pressure with high mechanical strength and low thermal conductivity. RSC Adv..

[10] Sui Z., Meng Q., Zhang X., Ma R., Cao B. (2012). Green synthesis of carbon nanotube-graphene hybrid aerogels and their use as versatile agents for water purification. J. Mater. Chem..

[11] Worsley M.A., Pauzauskie P.J., Olson T.Y., Biener J., Satcher J.H., Baumann T.F. (2010). Synthesis of Graphene Aerogel with High Electrical Conductivity. J. Am. Chem. Soc..

[12] Wu Z.-S., Sun Y., Tan Y.-Z., Yang S., Feng X., Müllen K. (2012). Three-Dimensional Graphene-Based Macro- and Mesoporous Frameworks for High-Performance Electrochemical Capacitive Energy Storage. J. Am. Chem. Soc..

[13] Xu Y., Sheng K., Li C., Shi G. (2010). Self-Assembled Graphene Hydrogel via a One-Step Hydrothermal Process. ACS Nano.

[14] Wu Z.-S., Yang S., Sun Y., Parvez K., Feng X., Müllen K. (2012). 3D Nitrogen-Doped Graphene Aerogel-Supported Fe_3_O_4_ Nanoparticles as Efficient Electrocatalysts for the Oxygen Reduction Reaction. J. Am. Chem. Soc..

[15] Kim T., Jung G., Yoo S., Suh K.S., Ruoff R.S. (2013). Activated Graphene-Based Carbons as Supercapacitor Electrodes with Macro- and Mesopores. ACS Nano.

[16] Jiang L., Lin B., Li X., Song X., Xia H., Li L., Zeng H. (2016). Monolayer MoS_2_–Graphene Hybrid Aerogels with Controllable Porosity for Lithium-Ion Batteries with High Reversible Capacity. ACS Appl. Mater. Interfaces.

[17] Chen Z., Ren W., Gao L., Liu B., Pei S., Cheng H.-M. (2011). Three-dimensional flexible and conductive interconnected graphene networks grown by chemical vapour deposition. Nat. Mater..

[18] Samad Y.A., Li Y., Schiffer A., Alhassan S.M., Liao K. (2015). Graphene Foam Developed with a Novel Two-Step Technique for Low and High Strains and Pressure-Sensing Applications. Small.

[19] Balandin A.A. (2011). Thermal properties of graphene and nanostructured carbon materials. Nat. Mater..

[20] Wicklein B., Kocjan A., Salazar-Alvarez G., Carosio F., Camino G., Antonietti M., Bergström L. (2015). Thermally insulating and fire-retardant lightweight anisotropic foams based on nanocellulose and graphene oxide. Nat Nano.

[21] Xie Y., Xu S., Xu Z., Wu H., Deng C., Wang X. (2016). Interface-mediated extremely low thermal conductivity of graphene aerogel. Carbon.

[22] Worsley M.A., Shin S.J., Merrill M.D., Lenhardt J., Nelson A.J., Woo L.Y., Gash A.E., Baumann T.F., Orme C.A. (2015). Ultralow Density, Monolithic WS_2_, MoS_2_, and MoS_2_/Graphene Aerogels. ACS Nano.

[23] Fang Y., Lv Y., Gong F., Elzatahry A.A., Zheng G., Zhao D. (2016). Synthesis of 2D-Mesoporous-Carbon/MoS_2_ Heterostructures with Well-Defined Interfaces for High-Performance Lithium-Ion Batteries. Adv. Mater..

[24] Li H., Yu K., Fu H., Guo B., Lei X., Zhu Z. (2015). Multi-slice nanostructured WS_2_@rGO with enhanced Li-ion battery performance and a comprehensive mechanistic investigation. Phys. Chem. Chem. Phys..

[25] Fan Z., Tng D.Z.Y., Nguyen S.T., Feng J.D., Lin C.F., Xiao P.F., Lu L., Duong H.M. (2013). Morphology effects on electrical and thermal properties of binderless graphene aerogels. Chem. Phys. Lett..

[26] Menzel R., Barg S., Miranda M., Anthony D.B., Bawaked S.M., Mokhtar M., Al-Thabaiti S.A., Basahel S.N., Saiz E., Shaffer M.S.P. (2015). Joule Heating Characteristics of Emulsion-Templated Graphene Aerogels. Adv. Funct. Mater..

[27] Cheng Y., Zhou S., Hu P., Zhao G., Li Y., Zhang X., Han W. (2017). Enhanced mechanical, thermal, and electric properties of graphene aerogels via supercritical ethanol drying and high-temperature thermal reduction. Sci. Rep..

[28] Fan Z., Tng D.Z.Y., Lim C.X.T., Liu P., Nguyen S.T., Xiao P., Marconnet A., Lim C.Y.H., Duong H.M. (2014). Thermal and electrical properties of graphene/carbon nanotube aerogels. Colloids Surf. A Physicochem. Eng. Asp..

[29] Hiramatsu M., Kondo H., Hori M., Gong J.R. (2013). Graphene Nanowalls. New Progress on Graphene Research.

[30] Zhang L., Chen G., Hedhili M.N., Zhang H., Wang P. (2012). Three-dimensional assemblies of graphene prepared by a novel chemical reduction-induced self-assembly method. Nanoscale.

[31] Patil S., Harle A., Sathaye S., Patil K. (2014). Development of a novel method to grow mono-/few-layered MoS2 films and MoS_2_-graphene hybrid films for supercapacitor applications. CrystEngComm.

[32] El-Mahalawy S.H., Evans B.L. (1977). Temperature Dependence of the Electrical Conductivity and Hall Coefficient in 2H-MoS_2_, MoSe_2_, WSe_2_, and MoTe_2_. Phys. Status Solidi B Basic Res..

[33] Marinho B., Ghislandi M., Tkalya E., Koning C.E., de With G. (2012). Electrical conductivity of compacts of graphene, multi-wall carbon nanotubes, carbon black, and graphite powder. Powder Technol..

[34] Yan R., Simpson J.R., Bertolazzi S., Brivio J., Watson M., Wu X., Kis A., Luo T., Hight Walker A.R., Xing H.G. (2014). Thermal Conductivity of Monolayer Molybdenum Disulfide Obtained from Temperature-Dependent Raman Spectroscopy. ACS Nano.

[35] Naidu G.A., Udo S. (2016). Thermal conductivity of bulk and monolayer MoS_2_. EPL (Europhys. Lett.).

[36] Liu J., Choi G.-M., Cahill D.G. (2014). Measurement of the anisotropic thermal conductivity of molybdenum disulfide by the time-resolved magneto-optic Kerr effect. J. Appl. Phys..

[37] Sahoo S., Gaur A.P.S., Ahmadi M., Guinel M.J.F., Katiyar R.S. (2013). Temperature-Dependent Raman Studies and Thermal Conductivity of Few-Layer MoS_2_. J. Phys. Chem. C.

[38] Rafiee M.A., Rafiee J., Wang Z., Song H., Yu Z.-Z., Koratkar N. (2009). Enhanced Mechanical Properties of Nanocomposites at Low Graphene Content. ACS Nano.

[39] Tang Z., Shen S., Zhuang J., Wang X. (2010). Noble-Metal-Promoted Three-Dimensional Macroassembly of Single-Layered Graphene Oxide. Angew. Chem..

[40] Shahil K.M.F., Balandin A.A. (2012). Graphene-Multilayer Graphene Nanocomposites as Highly Efficient Thermal Interface Materials. Nano Lett..

[41] Gong F., Bui K., Papavassiliou D.V., Duong H.M. (2014). Thermal transport phenomena and limitations in heterogeneous polymer composites containing carbon nanotubes and inorganic nanoparticles. Carbon.

[42] Gong F., Duong H.M., Papavassiliou D.V. (2015). Inter-Carbon Nanotube Contact and Thermal Resistances in Heat Transport of Three-Phase Composites. J. Phys. Chem. C.

[43] Fan Z., Gong F., Nguyen S.T., Duong H.M. (2015). Advanced multifunctional graphene aerogel—Poly(methyl methacrylate) composites: Experiments and modeling. Carbon.

[44] Zhang X., Sun D., Li Y., Lee G.-H., Cui X., Chenet D., You Y., Heinz T.F., Hone J.C. (2015). Measurement of Lateral and Interfacial Thermal Conductivity of Single- and Bilayer MoS_2_ and MoSe_2_ Using Refined Optothermal Raman Technique. ACS Appl. Mater. Interfaces.

[45] Yuan P., Li C., Xu S., Liu J., Wang X. (2017). Interfacial thermal conductance between few to tens of layered-MoS_2_ and c-Si: Effect of MoS_2_ thickness. Acta Mater..

